# Influence of divalent cations on the extraction of organic acids in coffee determined by GC-MS and NMR

**DOI:** 10.1016/j.heliyon.2024.e26625

**Published:** 2024-02-17

**Authors:** Tove Bratthäll, João Figueira, Malin L. Nording

**Affiliations:** a Department of Chemistry, Umeå University, 901 87, Umeå, Sweden; b Department of Chemistry, SciLife Lab, Umeå University, 901 87, Umeå, Sweden

**Keywords:** Coffee brewing, Magnesium, Calcium, GC-MS, NMR, Organic acid

## Abstract

The perceived flavor of coffee varies depending on the composition of the brewing water, and the influencing mechanisms are poorly understood. To investigate the effect of dissolved divalent cations on the extraction of organic acids in coffee, magnesium and calcium chloride salts were added pre- and post-brew. Citric, malic, lactic and quinic acid were analyzed using gas chromatography – mass spectrometry and nuclear magnetic resonance techniques. At concentrations typically found in drinking water, the salts resulted in limited variation of the acid content, while ten-fold higher salt concentrations produced more pronounced variations. Comparisons between pre- and post-brew additions showed similar acid content in most cases, suggesting that extraction of acids proceeds independent of the water composition. Interactions taking place post-brew may, however, influence the perceived flavor. A scientific basis for water quality recommendations in the coffee industry is long overdue and this work provides experimental and analytical contributions to continued research.

## Highlights

• Pre- and post-brew effects of divalent ions on organic acids in coffee were investigated.
• Regular levels of divalent ions displayed small effects on acid content in coffee.
• Ten-fold higher levels of divalent ions displayed larger effects on acid content in coffee.
• Extraction of acids in coffee seems to proceed independent of divalent ions.
• Perceived flavor of coffee is most likely due to interactions taking place post-brew.

## Graphical abstract

**Figure:**
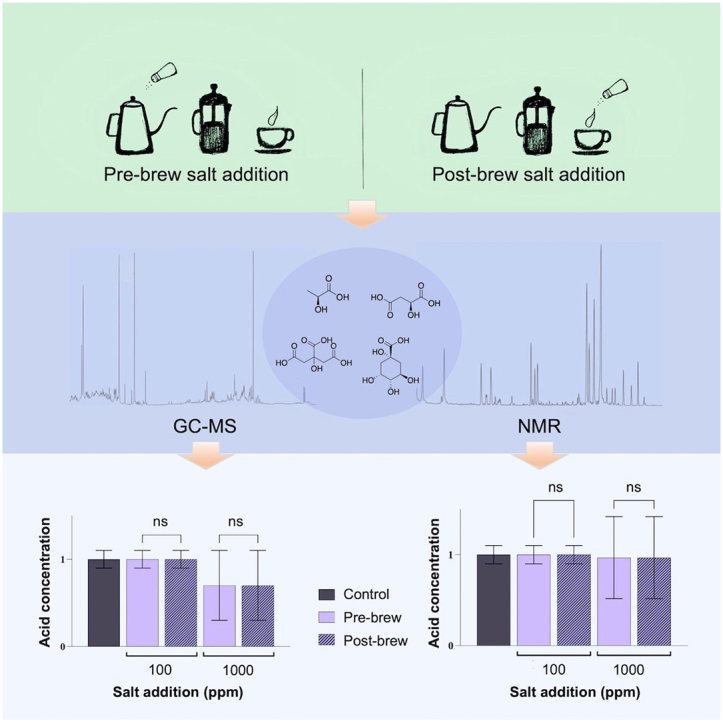

## 1 Introduction

Water is an essential ingredient in coffee brewing, making its quality a factor of high interest. It has been established that the perceived flavor of coffee is greatly affected by the water composition, and the matter has received substantial attention amongst professionals within the specialty coffee community [1]. Yet, it is one of the least studied parameters. The general recommendations are vague and lack a complete scientific foundation [1]. The Specialty Coffee Association of America suggests a total hardness between 50 and 175 ppm CaCO_3_, an alkalinity between 40 and 70 ppm CaCO_3_ and a pH near 7 for favorable extraction [2]. Note that hardness and alkalinity are described in ppm CaCO_3_ equivalents and not mass concentration [1]. Although the mechanisms of the carbonate system have been explored [[3], [4], [5]], the role of calcium and magnesium ions remains poorly understood.

The flavor of a coffee beverage is a combination of retro-nasal aroma impression and taste, caused by volatile and non-volatile flavor compounds present in the brew [6]. The chemical composition depends on a range of parameters, including species and origin of the green coffee bean [7,8], fermentation and drying processes [9], roasting degree [10], and the extraction process [6]. A good cup of coffee is characterized by a balance between aroma, acidity, bitterness, and astringency, accompanied by a pleasant mouthfeel [6]. Perceived acidity is believed to be caused by organic acids such as chlorogenic, citric, malic, lactic, and quinic acid [[11], [12], [13]] and to correlate to titratable acidity, although investigations have produced conflicting results [6,14,15]. Chlorogenic acids additionally contribute to astringency and, together with caffeine and trigonelline, to bitterness [6,13]. Bitterness is also largely contributed to by end products of reactions taking place during roasting [16].

The flavor compounds reach the cup through the solid-liquid extraction commonly referred to as brewing, and due to their varying solubility, these are extracted at different rates [6]. Highly polar compounds such as sugars and organic acids are rapidly extracted, while less soluble compounds, often associated with bitterness and astringency, require longer contact time with the brew water. Consequently, over-extraction is characterized by bitter and astringent flavors, while under-extraction results in a watery and acidic cup profile [16]. Parameters influencing the extraction include particle size [17], water temperature, contact time, coffee to water ratio [6] and water composition [3,4].

Small changes in the water composition substantially affect the cup quality [1], but whether the crucial differences arise during the extraction process, from interactions in the cup or with taste receptors is yet to be established. Carbonate rich water results in neutralization of acids, which, in addition to lowering the perceived acidity, increases the brewing time of espresso and drip coffee due to the resistance created by the release of carbon dioxide gas [[3], [4], [5], [6]]. Sodium softening involves the replacement of calcium and magnesium ions and is a technique used to reduce scale formation. Softened water additionally prolongs the brewing time, indicating the relevance of the cationic species present [5]. Further, computational modeling conducted by Hendon, Colonna-Dashwood & Colonna-Dashwood [18] suggested that divalent cations increase the extraction of flavor compounds such as organic acids. In contrast, a high mineral content has been shown to slow the extraction of tea components [[19], [20], [21]], as well as influence complexation phenomena in brewed tea [22]. The flavors of divalent salts are complex, and the underlying mechanisms for how these are perceived are poorly understood. Chloride salts of magnesium and calcium are primarily characterized by saltiness and bitterness, and larger anions such as lactate have been found to suppress the perceived taste of the cations [23]. The concentrations at which minerals become perceivable in coffee (300 ppm Ca^2+^, 200 ppm Mg^2+^) are, however, typically not reached in tap water [24]. Despite being present at sub-threshold levels, calcium and magnesium have been found to affect the perceived sweetness of sweeteners [25], and Fekete [26] recently raised the question whether the cations primarily influence flavor perception rather than coffee extraction.

To study the link between water quality and flavor perception, chemical analysis of the coffee constituents is necessary. Analytical methods commonly used for characterization of the chemical composition in coffee include high performance liquid chromatography (HPLC), nuclear magnetic resonance (NMR) and gas chromatography-mass spectrometry (GC-MS) techniques [6,10,15,27]. While GC-MS is typically used for analysis of volatile compounds (e.g. coffee aroma), it has also been used to study organic acids in for example wine and saffron [28,29].

The present study aimed to explore the mechanisms responsible for the influence of water quality on coffee flavor by investigating the effect of dissolved magnesium and calcium ions on the extraction of quinic, lactic, malic, and citric acid in the coffee brew. Two analytical methods for the acids, GC-MS and NMR, were employed. To address limitations of the latter, the impact of salt concentration on NMR results was examined. To our knowledge, this is the first study designed to experimentally investigate the effect of dissolved cations on the extraction of coffee acids relevant for flavor perception.

## 2 Materials and methods

### 2.1 Coffee beans and preparation

Zoega's Selected Presso, a dark-roasted blend of 100% Arabica beans from Kenya, Latin America and Brazil, coarsely ground for French press, was chosen for the extractions. Brewing was performed according to the Specialty Coffee Association standard protocol using 11.00 g of coffee and 200 (±1) mL of water heated to 93 °C and steeped for 5 min [2]. The brew was vacuum filtrated through a Munktell filter paper (grade 3).

### 2.2 Experimental design

**Fig. 1:**
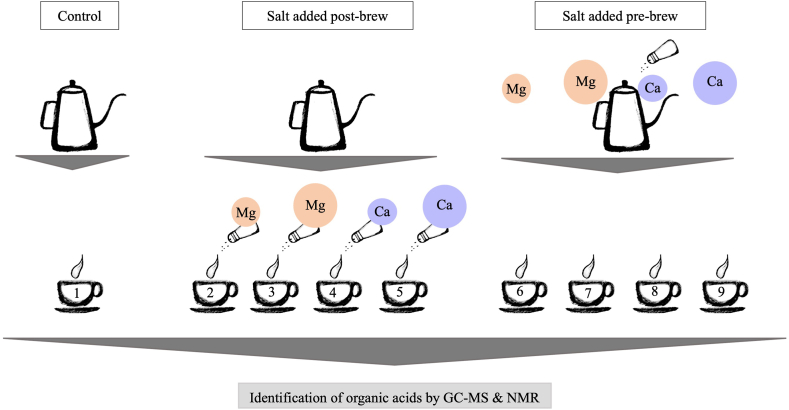
Illustration of the experimental design. The control sample (1) was brewed with pure water. The samples in the post-brew group were brewed with pure water, followed by salt additions to final concentrations of 100 ppm MgCl_2_ (2), 1000 ppm MgCl_2_ (3), 100 ppm CaCl_2_ (4) and 1000 ppm CaCl_2_ (5). The pre-brew group was brewed using prepared stock solutions of 100 ppm MgCl_2_ (6), 1000 ppm MgCl_2_ (7), 100 ppm CaCl_2_ (8) and 1000 ppm CaCl_2_ (9).

The coffee was prepared with varying concentrations of calcium chloride (CaCl_2_) and magnesium chloride (MgCl_2_) added pre- or post-brew, as illustrated in Fig. 1. For post-brew addition, reagent grade MgCl_2_·6H_2_O and CaCl_2_·2H_2_O were dissolved in Milli-Q water to concentrations of 20% (Supplementary Table S1) and added to brewed coffee to reach final concentrations of 100 and 1000 ppm. For pre-brew addition, four stock solutions containing 100 ppm and 1000 ppm of each salt were prepared (Supplementary Table S2).

### 2.3 Analysis of compounds

#### 2.3.1 GC-MS

The samples were prepared for GC-MS analysis by adding 900 μL extraction mixture (methanol/water, 90/10, v/v) with internal standards to 100 μL brewed coffee. Succinic acid-D4, Salicylic acid-D6, L-Glutamic acid-13C5,15 N, Putrescine-D4, D-Glucose-13C6, Hexadecanoic acid-13C4, Sucrose-13C12 purchased from Sigma (St. Louis, MO, USA) and L-Proline-13-C5, Di-Na-alpha-ketoglutarate-13C4, Myristic acid-13C3 and Cholesterol D7 purchased from Cil (Andover, MA, USA) were used as internal standards. The mixture was cooled and centrifuged at 13500 rpm for 10 min at +4 °C, followed by removal of the supernatant. 100 μL of each sample was transferred to a GC vial, solvents were evaporated, and the extracts were stored at −20 °C.

Derivatization of the dried extracts was initiated by the addition of 30 μL methoxyamine in pyridine (15 μg/μL) followed by 10 min in a vortex mixer. The samples were left in room temperature overnight prior to the addition of 60 μL of a mixture containing 30 μL of N-Methyl-N-(trimethylsilyl)trifluoroacetamide (MSTFA) with 1% trimethylsilyl chloride (TMCS), and 30 μL methyl stearate in heptane (15 ng/μL), vortexed and left in room temperature for one (1) hour. An L-PAL3 autosampler (CTC Analytics AG, Switzerland) was employed for the injection of 1 μL of the sample into an Agilent 7890B gas chromatograph using a splitless mode and an injection temperature of 270 °C. The purge was turned on after 60 s with a 20 mL/min flow rate. Separation of analytes was carried out on a Rxi-5 Sil MS capillary column (10 m × 0.18 mm x 0.18 μm thickness, Restek Corporation, USA) with a low-polarity crossbond 1,4-bis(dimethylsiloxy)phenylene dimethyl polysiloxane stationary phase with a carrier gas flow of 1 mL/min. The oven temperature was kept at 70 °C for 2 min, then ramped to 320 °C at a rate of 40 °C/min and maintained for 2 min. The effluent was transferred to the ion source of a Pegasus BT GC-TOF-MS (Leco Corp., St Joseph, MI, USA) through a heated transfer line (250 °C). The TOF-MS was operated with electron ionization (70 eV, 2 mA current), a source temperature of 200 °C and a detector voltage of 1800–2300 V. 30 spectra/s of ions in the mass range of 50–800 *m*/*z* were recorded after a solvent delay of 150 s.

GC-MS data files were exported in NetCDF format from the ChromaTOF software and preprocessing of the raw data, including baseline correction, spectral alignment, data compression and Multivariate Curve Resolution, was performed in MATLAB R2016a (Mathworks, Natick, MA, USA). Identification of the mass spectra was achieved by comparing the obtained mass spectra and retention time index to those of libraries using the NIST MS 2.0 software and an in-house database (Swedish Metabolomics Center). n-Alkanes (C8–C40) were used for retention-index conversion of the timeline and semi-quantitative results were obtained based on internal standard concentrations.

#### 2.3.2 NMR

Samples for NMR analysis were prepared by centrifuging 1 mL of each coffee sample. 300 μL of the supernatant was mixed with 300 μL phosphate buffer (pH 6) and 10% D_2_O containing 15.72 mM 3-(trimethylsilyl)-2,2,3,3-tetradeuteropropionic acid (TSP) and transferred to a 5 mm Samplejet rack tube. TSP was used as a concentration and chemical shift (*δ*_H_ = 0 ppm) internal reference. Samples were kept at 6 °C until analyzed, at which point the temperature was equilibrated at 25 °C for 5 min before any acquisition was done. The analysis was performed using a Bruker 600 MHz Avance III HD spectrometer equipped with a 5 mm BBO cryoprobe with z‐gradients (Bruker Biospin, Rheinstetten, Germany). A standard NOESY 1Dpresat (nuclear Overhauser effect spectroscopy pulse sequence; noesygppr1d; Bruker BioSpin), using 92 scans, 98304 data points, a spectral width of 18028 Hz, an acquisition time of 2.7 s, a relaxation delay of 4 s and a mixing time of 0.01 s, was applied to all samples. An extra ^1^H–^1^H TOCSY (Total Correlation Spectroscopy) experiment was also performed on sample 1.1 to help elucidate metabolite assignment. Acquisition and pre-processing of the spectra was performed on TopSpin 3.6.1 and 3.6.2 respectively. Chenomx NMR Suite 3.8 was used for fitting, quantification, and assignment.

Further NMR experiments were performed to investigate the influence of salt addition on NMR quantification, described in the Supplementary Material.

### 2.4 Statistical analysis

The results were expressed as mean values and standard deviation (n = 5 per group). One-way ANOVA was performed to identify statistically significant (*p* < 0.05) differences between samples, followed by Fisher's LSD test using GraphPad Prism 9.2.0 (GraphPad Software, San Diego, CA, USA).

## 3 Results and discussion

**Fig. 2:**
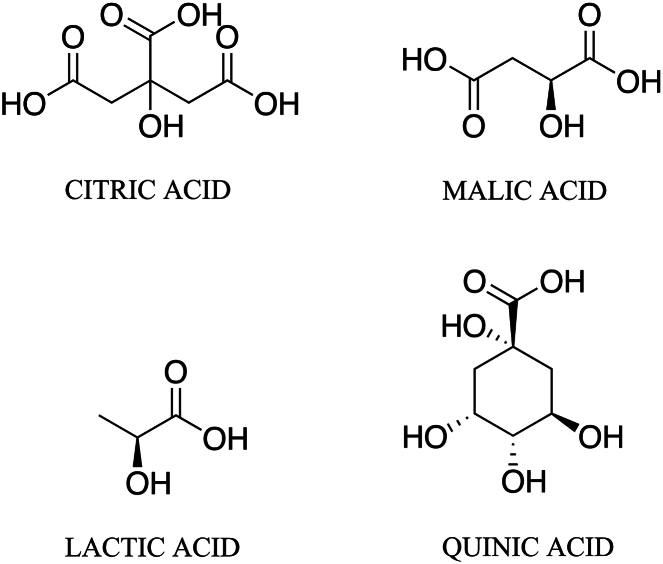
Organic acids present in coffee selected for analysis by GC-MS and NMR.

**Fig. 3:**
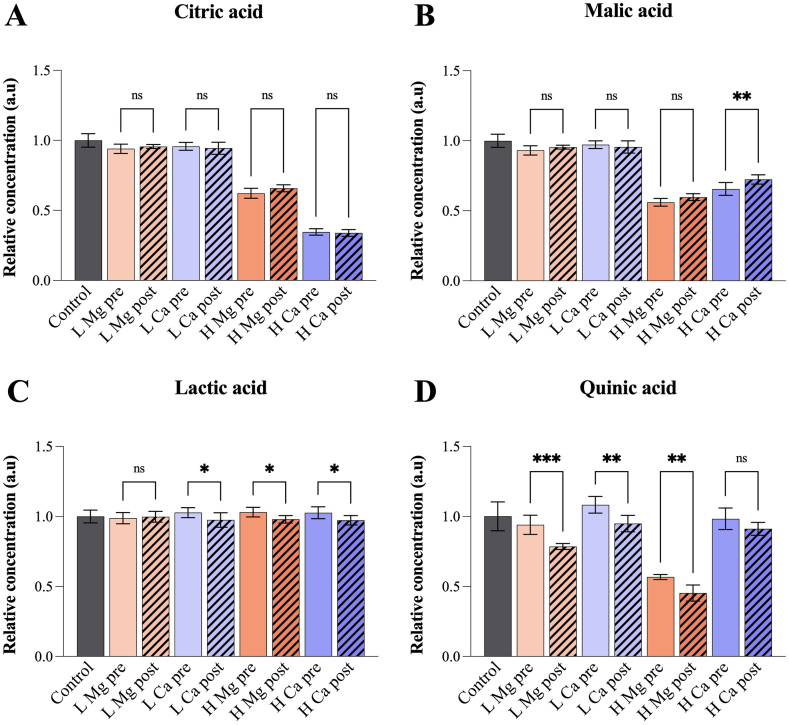
Relative concentration of selected acids as measured by GC-MS. The uncertainties are represented by the standard deviation of the mean (n = 5). Low (100 ppm) and high (1000 ppm) salt concentrations are abbreviated L and H, respectively. Significant difference between pre- and post-brew addition of salts is indicated (*p < 0.05; **p < 0.01; ***p < 0.001; ns = no significant difference).

**Fig. 4:**
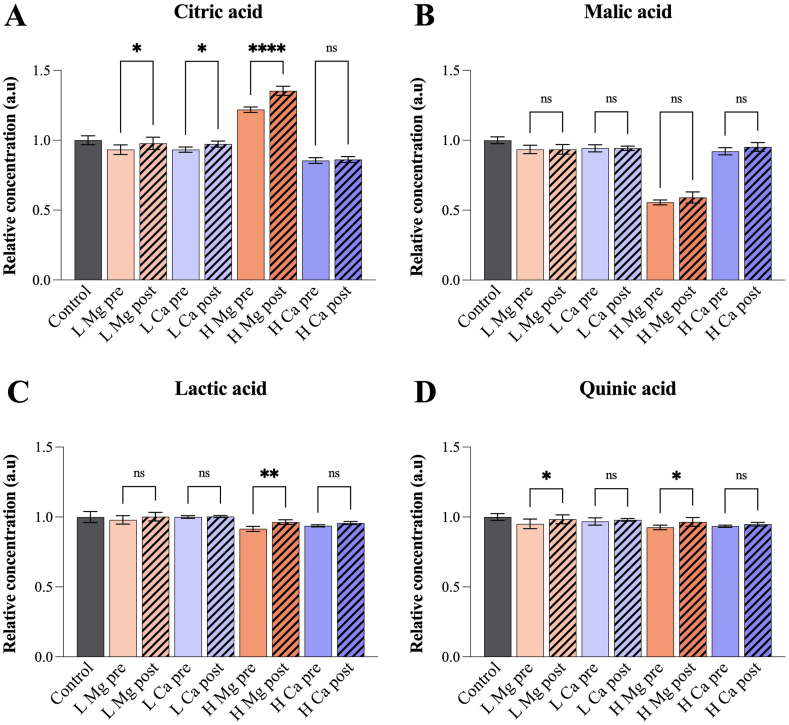
Relative concentration of selected acids as measured by NMR. The uncertainties are represented by the standard deviation of the mean (n = 5). Low (100 ppm) and high (1000 ppm) salt concentrations are abbreviated L and H, respectively. Significant difference between pre- and post-brew addition of salts is indicated. (*p < 0.05; ****p < 0.0001; ns = no significant difference).

To address the objective of investigating the effects of salt addition on extraction, salts were added pre- and post-brew. Quinic acid, lactic acid, malic acid and citric acid (Fig. 2), selected based on their flavor properties, were detected in all samples. For a representative GC-MS chromatogram and NMR spectra, see Supplementary Figs. S1 and S2. While previous *in silico* studies suggest that salts enhance the extraction of acids [18], the highest acid concentrations in the present study were generally found in the control group, and differences between pre- and post-brew additions were in most cases not significant (Fig. 3, Fig. 4). Salts added at 100 ppm had slightly reducing or no effect on the acid content (Supplementary Figs. S3–S4) and similar results were obtained from NMR and GC-MS investigations. Salts added at 1000 ppm produced substantial variation in the concentrations of quinic, malic, and citric acid and the variation differed in terms of degree and direction depending on the acid and the analytical method used (Supplementary Figs. S3–S4).

### 3.1 Pre-brew compared to post-brew addition of salts

Comparison of the acid concentrations with respect to pre- and post-brew salt addition are shown in Fig. 3, Fig. 4. Although small but significant differences were observed, there was no clear trend regarding whether pre- or post-brew addition caused the lower concentrations compared to the control group. Based on GC-MS analysis, there was no significant difference in the citric acid concentration with respect to pre-compared to post-brew addition of salt (Fig. 3A), and the only significant difference for malic acid was found after addition of 1000 ppm CaCl_2_ (Fig. 3B). For lactic acid, all post-brew salt additions, except 100 ppm MgCl_2_, resulted in slightly lower concentrations compared to pre-brew addition (Fig. 3C). The differences were, however, not significant when compared to the control group (Supplementary Fig. S3). Quinic acid was significantly lower after all post-brew salt additions, except for 1000 ppm CaCl_2_ (Fig. 3D). As further discussed in 3.3, the observed reductions are most likely a result of interactions taking place after, rather than during, the extraction process.

NMR analysis indicated slightly lower citric acid concentrations for pre-compared to post-brew additions of salts at 100 ppm, and that pre-brew addition of 1000 ppm MgCl_2_ increased the concentration of citric acid to a lesser extent than post-brew addition (Fig. 4A). There was no significant difference in citric acid concentration between pre- and post-brew addition of 1000 ppm CaCl_2_. For malic, lactic and quinic acid, pre- and post-brew comparisons generally resulted in no significant differences (Fig. 4B–D). The exceptions were lactic acid with 1000 ppm MgCl_2_ and quinic acid with 100 ppm and 1000 ppm MgCl_2_, where slightly lower concentrations were found after pre-brew addition (Fig. 4C–D). The similarity between samples with pre- and post-brew additions of salt reduces the likelihood of the cations affecting the extraction of organic acids, and rather suggests that whatever interactions may take place are more likely to occur in the final cup than in the coffee bed during extraction.

The findings contradict previously suggested mechanisms for the role of calcium and magnesium in extraction of acids. Hendon et al. [18] investigated the thermodynamic binding energies of selected organic acids, caffeine and eugenol to sodium, magnesium, and calcium ions, and found that dissolved cations interact with the nucleophilic motifs of solvated coffee constituents. It was concluded that the extraction of organic flavor compounds in coffee is enhanced by the cations and that magnesium-rich water has the most extracting ability [18]. As organic acids are polar compounds and highly soluble in water, they are extracted quickly during brewing, making it rather unlikely that their extraction is facilitated by ions. It is a reasonable conclusion that all acids available for extraction are removed from the ground coffee in the early stages of brewing, independent of the water composition, and that the cations are more likely to aid the extraction of, if anything, compounds of lower solubility such as phenylindanes and chlorogenic acid lactones [11,16]. The theory suggested by Fekete [26] regarding cations influencing perception rather than extraction of flavorsome compounds, which is supported by the data obtained in the present study, was based on a sensory experiment with calcium and magnesium chloride added pre- and post-brew, where the tasting panel determined there was no difference in perceived flavor.

### 3.2 Effects of 100 ppm salt in comparison to no salt (control)

Upon comparison of the 100 ppm groups to the control group, similar or higher acid concentrations were found in the latter (*p* < 0.05), except for quinic acid with pre-brew addition of CaCl_2_ measured by GC-MS (Supplementary Figs. S3–S4). The largest decrease was observed for quinic acid (around 20%) after post-brew addition of MgCl_2_ (Supplementary Fig. S3). Although statistical investigations of the NMR data produced similar results to GC-MS, discrepancies were observed, partly due to larger standard deviations associated with the GC-MS data. Notably, quinic acid did not display a comparable variation according to the NMR results.

### 3.3 Effects of 1000 ppm salt in comparison to no salt (control)

The data obtained from GC-MS analysis indicated that 1000 ppm MgCl_2_ significantly reduced the concentrations of citric, malic and quinic acid by 40–60% (Supplementary Fig. S3). 1000 ppm CaCl_2_ resulted in concentration decreases of citric and malic acid of 70% and 25–40%, respectively. Quinic acid was slightly reduced by post-brew addition of 1000 ppm CaCl_2_. No significant variation was detected in the lactic acid concentration. The NMR investigations indicated that MgCl_2_, in accordance with GC-MS data, caused a 40% reduction of malic acid, whereas citric and quinic acid did not undergo comparable decreases (Supplementary Fig. S4). Surprisingly, the citric acid concentration measured by NMR increased by 20% and 30–40% in samples with pre- and post-brew addition of MgCl_2_, respectively. Lactic and quinic acid decreased slightly in samples with both pre- and post-brew addition of both salts. 1000 ppm CaCl_2_ reduced the citric and malic acid content to a lesser extent in NMR than GC-MS measurements.

The observed reductions in acid concentrations could be explained by the cations forming complexes with the acids, allowing them to escape detection. In milk, soluble calcium is partly present as complexes with citrate, and models for the ion equilibria have been shown to apply to magnesium as well [30]. Metal ions are known to influence detected polyphenol concentrations in herbal extracts due to complexation [31], and phenomena such as tea scum formation, and absence thereof, in tea infusions have been attributed to complexation involving calcium, carbonate, organic matter and citrate [22]. Studies suggesting slowed extraction of tea components by high mineral content in the brew water, have attributed the results to complexation and subsequent retention of compounds in the cell walls [[19], [20], [21]]. As the physicochemical properties of coffee and tea differ substantially, direct comparisons to the present study cannot be made. The results do, however, warrant investigations regarding the role of complexation phenomena in brewed coffee. As complexes largely depend on environmental factors such as pH [32], temperature [33], and solvent [31], and as sample preparation protocols differ for GC-MS and NMR analysis, this could partly explain the discrepancies between the GC-MS and NMR data. For example, coffee typically has a pH between 5 and 6 [6], and while it was brought to pH 6 prior to NMR analysis (through addition of phosphate buffer), the preparation for GC-MS analysis involved a ten-fold dilution with methanol affecting pH of the solvent and pK_a_-values of the acids [34]. In the case of GC-MS, the observed losses could have occurred during sample extraction, derivatization, or analysis. In the case of NMR, biased results are likely due to interference from the salts affecting spectral line shape and chemical shift (see section 3.4). For accurate interpretation of the effects of 1000 ppm salt additions, further research regarding complex formation in brewed coffee and its dependence on sample preparation (e.g. extraction solvent and buffer) and detection method is required.

### 3.4 Limitations of the study

This study was exclusive of other ions normally present in natural waters such as bicarbonate. As the aim was to investigate the sole effect of the selected cations, this was a deliberate choice. Nevertheless, repeating the experiment with the addition of carbonate species would most likely produce different results. For example, the cations might indirectly affect flavor by governing the activity of the carbonate ion. Research by Navarini and Rivetti [5] suggests that the activity of carbonate depends on the presence of calcium and magnesium ions, as the mineral replacement associated with sodium softening was considered more relevant for prolonged brewing time than the overall bicarbonate content.

Extrapolation of the findings would entail consideration of factors such as brewing method, coffee, or analytical method. In filter or espresso brewing, indirect effects on extraction, such as prolonged brewing time, could be evident. As the chemical composition of coffee varies with factors such as origin and roasting degree, the interactions taking place might vary accordingly. Although producing consistent results at low salt concentrations, GC-MS and NMR data were inconclusive at high salt concentrations, attenuating the need for attention to water quality when preparing coffee for analytical measurements. This does, however, not negate the similarities between pre- and post-brew additions. It further emphasizes the importance of investigating complexation phenomena during sample preparation procedures that might affect the results.

To address these limitations, additional NMR experiments using pure acid solutions, as outlined in Supplementary material, were performed to ascertain if quantification of the acids was influenced by salt addition. Higher concentrations (1000 ppm) of MgCl_2_ and CaCl_2_ produced signal broadening and a marked chemical shift difference compared to the control (Supplementary Fig. S5), possibly due to chelation of Mg^2+^ and Ca^2+^ by citrate and malate. Both organic acids are known to chelate divalent cations [[35], [36], [37]]. Addition of lactate suppressed the effects of the salts (data not shown), implying that a coffee beverage, containing a wide range of organic acid and other constituents, will facilitate interactions that influence quantification results. Both overestimation and underestimation of the acid content were observed, emphasizing the need for caution when interpreting quantitative data.

Interpretations of the study are limited by the lack of scientific literature regarding the sensory effects of minerals. Often cited early work by Lockhart [24] and Pangborn [38] suggest that ions must be present above certain threshold concentrations in order to produce detectable effects, and that coffee prepared with distilled water and, even more so, with high concentrations of CaCl_2_ and MgCl_2_, is excessively sour, while water rich in carbonate and bicarbonate produce bitter and flat coffee. Nowadays, it is recognized that even small changes in water composition substantially affect the cup quality [1], and perceived differences are likely to be more pronounced due to general improvements along the coffee production chain. Light roasted specialty coffee highlighting the characteristics of different origins and species has become increasingly popular in the last decades, combined with development of professional coffee tasters, barista competitions and tasting protocols. Science has, however, not kept up, and more recent studies merely correlates increased total hardness and alkalinity to reduced intensity of nearly all coffee attributes, including acidity and bitterness [1]. Within the coffee community, experiments conducted by professionals in the industry are, despite not being reviewed or published according to scientific standard, generally accepted. Consequently, a substantial gap has developed between the coffee community and the scientific community. To determine the potential relevance of complexation between cations and acids for coffee flavor, a sensory experiment clearly identifying perceived differences between magnesium and calcium addition in combination with chemical analysis of acids believed to influence flavor, is desirable.

## 4 Conclusion

In this study, an experiment was designed to evaluate the effect of divalent cations on the extraction of selected organic acids in coffee. Experimental data implied that dissolved magnesium and calcium chloride salts did not affect the extraction of the acids, yielding the conclusion that the extraction proceeds independent of the water composition. The salts did, however, influence the analytical results, regardless of being added pre- or post-extraction. Salts added at concentrations typical for drinking water generated either no change or slight reductions in acid contents, while ten-fold higher salt concentrations resulted in large variations and caused GC-MS and NMR to produce substantially different results. Additional NMR experiments revealed substantial influence of high salt content on spectral line shape and chemical shift. Little is known about complexation phenomena involving cations and coffee constituents and the potential impact on detection and quantification by various analytical methods, prompting further investigations. Repeating the experiment with focus on less soluble analytes than organic acids could provide a more complete picture. Moreover, complete sensory evaluations of coffee prepared with different water qualities, exclusively focused on cations, and in combination with chemical analyses of the acid content are warranted.

## Funding

This research did not receive any specific grant from funding agencies in the public, commercial, or not-for-profit sectors.

## CRediT authorship contribution statement

**Tove Bratthäll:** Writing – original draft, Visualization, Methodology, Investigation, Formal analysis, Data curation, Conceptualization. **João Figueira:** Validation, Software, Formal analysis, Data curation, Conceptualization, Writing – review & editing, Visualization, Resources, Methodology. **Malin L. Nording:** Conceptualization, Writing – review & editing, Supervision, Resources, Project administration, Methodology, Investigation.

## Declaration of competing interest

The authors declare that they have no known competing financial interests or personal relationships that could have appeared to influence the work reported in this paper.
