# Supplementary material for: Influence of divalent cations on the extraction of organic acids in coffee determined by GC-MS and NMR
Source: Heliyon. 2024 Feb 17;10(5):e26625. doi: 10.1016/j.heliyon.2024.e26625 (PMC10907646; doi:10.1016/j.heliyon.2024.e26625)
Supplement: Multimedia component 1 [file mmc1.docx]

**Supplementary Material**

Influence of divalent cations on the extraction of organic acids in coffee determined by GC-MS and NMR

Tove Bratthälla, João Figueira^b^, Malin L. Nording^a,*^

^a^Department of Chemistry, Umeå University, 901 87 Umeå, Sweden

^b^Department of Chemistry, SciLife Lab, Umeå University, 901 87 Umeå, Sweden

**Influence of high salt concentration on NMR quantification**

Further NMR experiments were performed to ascertain how NMR quantification could be influenced by the addition of salt solutions. Similarly to the coffee samples, samples were prepared with 300 µL of phosphate buffer, 100 µL of a citric and malic solution (0.50 and 2.08 mM respectively), 10 µL TSP (53.2 mM in D_2_O), 40 µL miliQ Water and 50 µL D_2_O; Depending on what was being tested, 100 µL CaCl_2_ or Mg Cl_2_ (0.28 or 0.028 % m/v) was then added. For the control, no Ca/Mg Cl_2_ solution was added and instead the total volume of miliQ water was 140 µL. NMR experiments also followed the same method as described for the coffee samples.

Addition of the higher concentration Mg and CaCl_2_ solutions produces a marked chemical shift difference from the control (Figure S5). For example from 2.354 to 2.377 ppm, for malic acid when treated with the 1000 ppm MgCl_2_ solution. The effect is also visible for the citrate peaks or when using CaCl_2_ instead.

The chemical shift differences may be the result of the chelation of Mg^2+^ and Ca^2+^ by citrate and malate. Both organic acids are known to chelate divalent cations (Karipides, 1979; Herdtweck et al., 2011; Kaduk, 2020a; Kaduk, 2020b)

Although no reports are available for the ^1^H chemical shifts of these specific chelated species, a thorough report (Hafer et al., 2020) on chelation of several divalent cations (Be^2+^, Mg^2+^, Ca^2+^, Sr^2+^, Zn^2+^, Cd^2+^, Hg^2+^, Sn^2+^, and Pb^2+^) by EDTA (ethylenediaminetetraacetic acid) shows the chemical shift change for the ^1^H signals of EDTA when in its metal complex form as a function of the cation. Furthermore, the authors also report signal broadening that arises from averaging of the signals arising from the complex and non-complex EDTA species. In fact, this could very well be the case for the broadening of the citrate and malate signals when higher concentrations of Mg^2+^/Ca^2+^ are present in solution. It was observed for the fresh test samples with high Mg^2+^/Ca^2+^ concentrations (not shown) and will undoubtedly influence NMR quantification. The extension of the quantification differences for the coffee solutions treated with high concentration Mg^2+^/Ca^2+^ solutions, i.e., the coffee samples sample that were treated with Mg2+ solution suffered higher quantification differences than those treated with Ca2+. Differet chelation effects for Mg^2+^ and Ca^2+^ were reported for in prednisolone (Carillo et a., 2019)

**References**

E. Hafer, U. Holzgrabe, K. Kraus, K. Adams, J. M Hook, J. M., Dieh, B. Qualitative and quantitative 1H NMR spectroscopy for determination of divalent metal cation concentration in model salt solutions, food supplements, and pharmaceutical products by using EDTA as chelating agent, *Magn. Reson. Chem.*, 58 (2020) 653–665, https://doi.org/10.1002/mrc.5009

E. Herdtweck, T. Kornprobst, R. Sieber, L. Straver, J. Plank, Crystal Structure, Synthesis, and Properties of tri-Calcium di-Citrate tetra-Hydrate [Ca_3_(C_6_H_5_O_7_)_2_(H_2_O)_2_]·2H_2_O, *Z. Anorg. Allg. Chem.,* 637 (2011) 655–659, https://doi.org/10.1002/zaac.201100088

K. D. Carillo, D. Wuc, S-C. Lin, S-L. Tsai, J-J. Shie, D-L. Tzouc, D-L. M. Magnesium and calcium reveal different chelating effects in a steroid compound: A model study of prednisolone using NMR spectroscopy, *Steroids*, 150 (2019) 108429, https://doi.org/10.1016/j.steroids.2019.108429

J. A. Kaduk, Crystal structure of aqua(citric acid)(hydrogencitrato)calcium monohydrate, [Ca(HC6H5O7)-(H3C6H5O7)(H2O)].H2O, from synchrotron X-raypowder data, and DFT-optimized crystal structure of existing calcium hydrogen citrate trihydrate,[Ca(HC6H5O7)(H2O)3], *Acta Crystallogr.*, E76 (2020a) 1689–1693, https://doi.org/10.1107/S2056989020012864

J. A. Kaduk, Crystal structures of two magnesium citrates from powder diffraction data, *Acta Crystallogr. E: Crystallogr. Commun.*, 76(10) (2020b) 1611–1616, <https://doi.org/10.1107/S2056989020011913>

A. Karipides, Crystal structure of ((S)-malato)tetraaquamagnesium(II) hydrate. Versatility of (S)-malate-metal ion binding, *Inorg. Chem.*, 18(11) (1979) 3034–3037, https://doi.org/10.1021/ic50201a018

**Supplementary Tables**

**Table S1.** Preparation of salt solutions used for post-brew addition.

| Sample | Solution | Reagent added (g/L)^1^ | | (mol/L) |  | Volume added (uL)^2^ | |
| --- | --- | --- | --- | --- | --- | --- | --- |
|  |  | MgCl_2_·6H_2_O | CaCl_2_·2H_2_O |  |  | 100 ppm | 1000 ppm |
| 2,3  4,5 | 20% MgCl_2_  20% CaCl_2_  20% MgCl_2_  20% CaCl_2_ | 441  -  Mg^2+^ | -  265  Ca^2+^ | 2.169  1.802 |  | 25  25 | 250  250 |
|  |  | 51.0  - | -  72.2 |  |  |  |  |

^1^Reagent grade chemicals were added to ultra-pure water.

^2^To 50 mL brewed coffee.

**Table S2.** Preparation of salt solutions used for pre-brew addition.

| Sample | Solution | Reagent added (mg/L)^1^ | | (mmol/L) | | Total hardness^2^ |
| --- | --- | --- | --- | --- | --- | --- |
|  |  | MgCl_2_·6H_2_O | CaCl_2_·2H_2_O | |  |  |
| 6  7  8  9 | 100 ppm MgCl_2_  100 ppm CaCl_2_  1000 ppm MgCl_2_  1000 ppm CaCl_2_  100 ppm MgCl_2_  100 ppm CaCl_2_  1000 ppm MgCl_2_  1000 ppm CaCl_2_ | 220  -  2200  -  Mg^2+^ | -  132  -  1320  Ca^2+^ | | 1.1  0.7  10.8  6.8 | 105.1  90.2  1051  902 |
|  |  | 25.5  -  255  - | -  36.1  -  361 | |  |  |

^1^Reagent grade chemicals were added to ultra-pure water.

^2^Expressed as ppm CaCO_3_.

**Supplementary Figures**


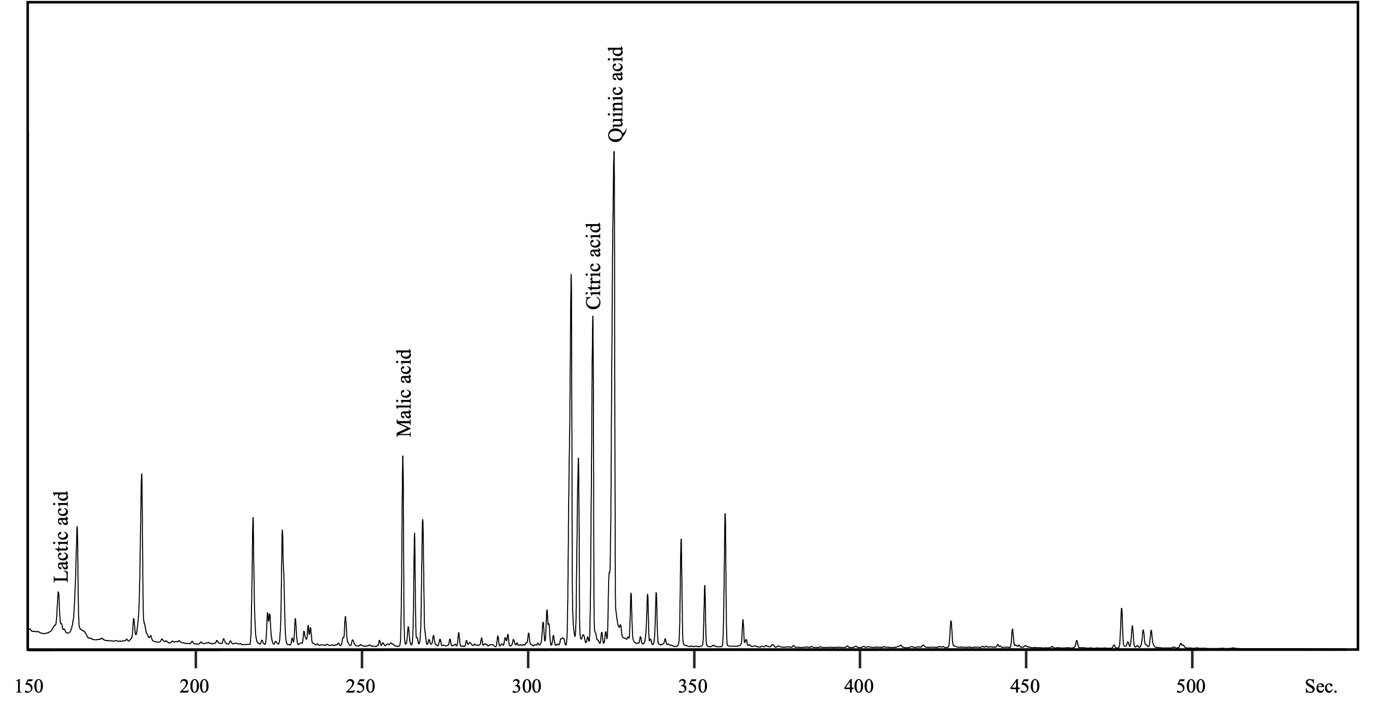


**Figure S1.** Representative GC-MS chromatogram for one of the analyzed coffee samples.


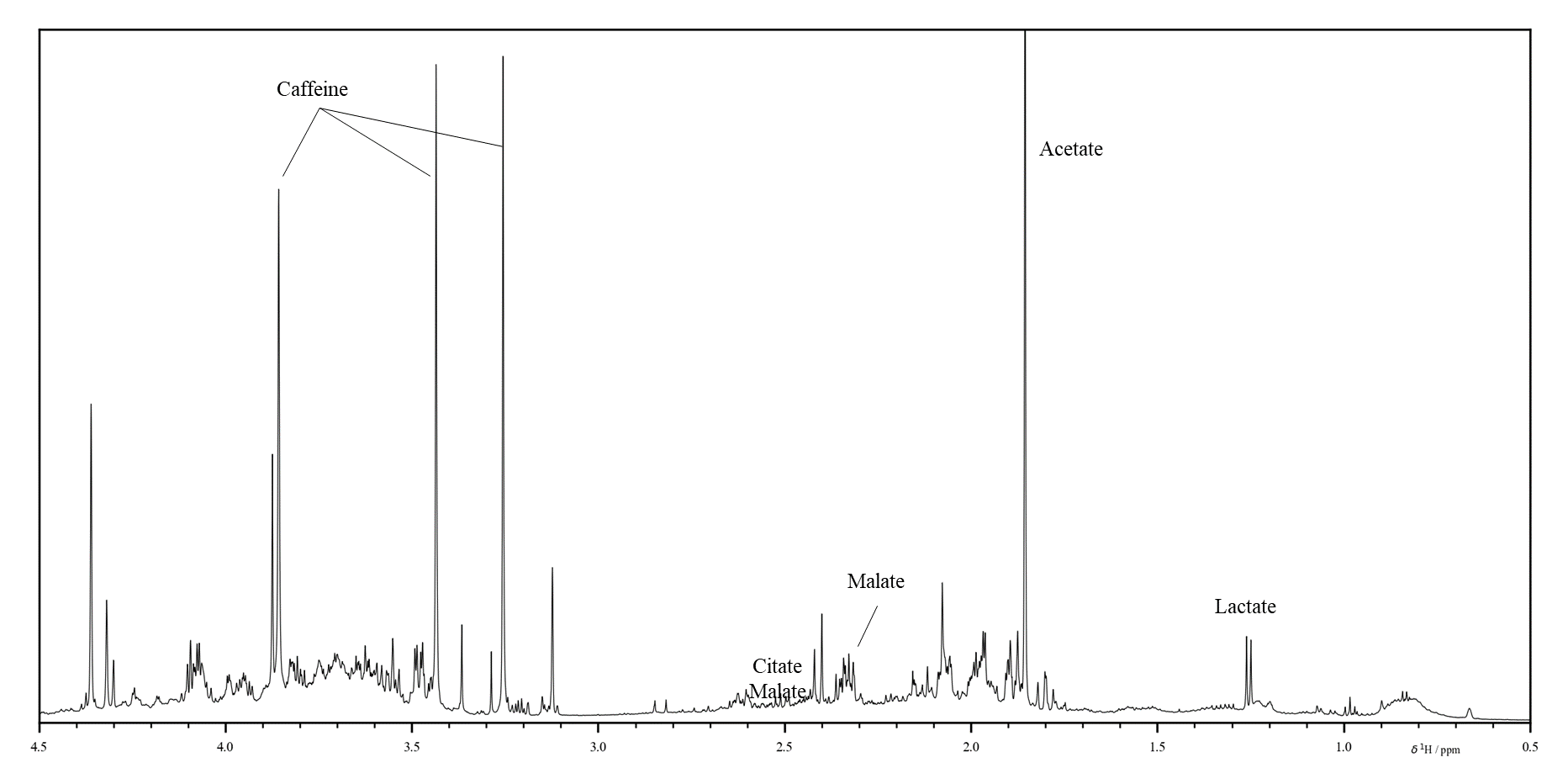


**Figure S2.** Representative ^1^H NMR spectral region for one of the analyzed coffee samples.


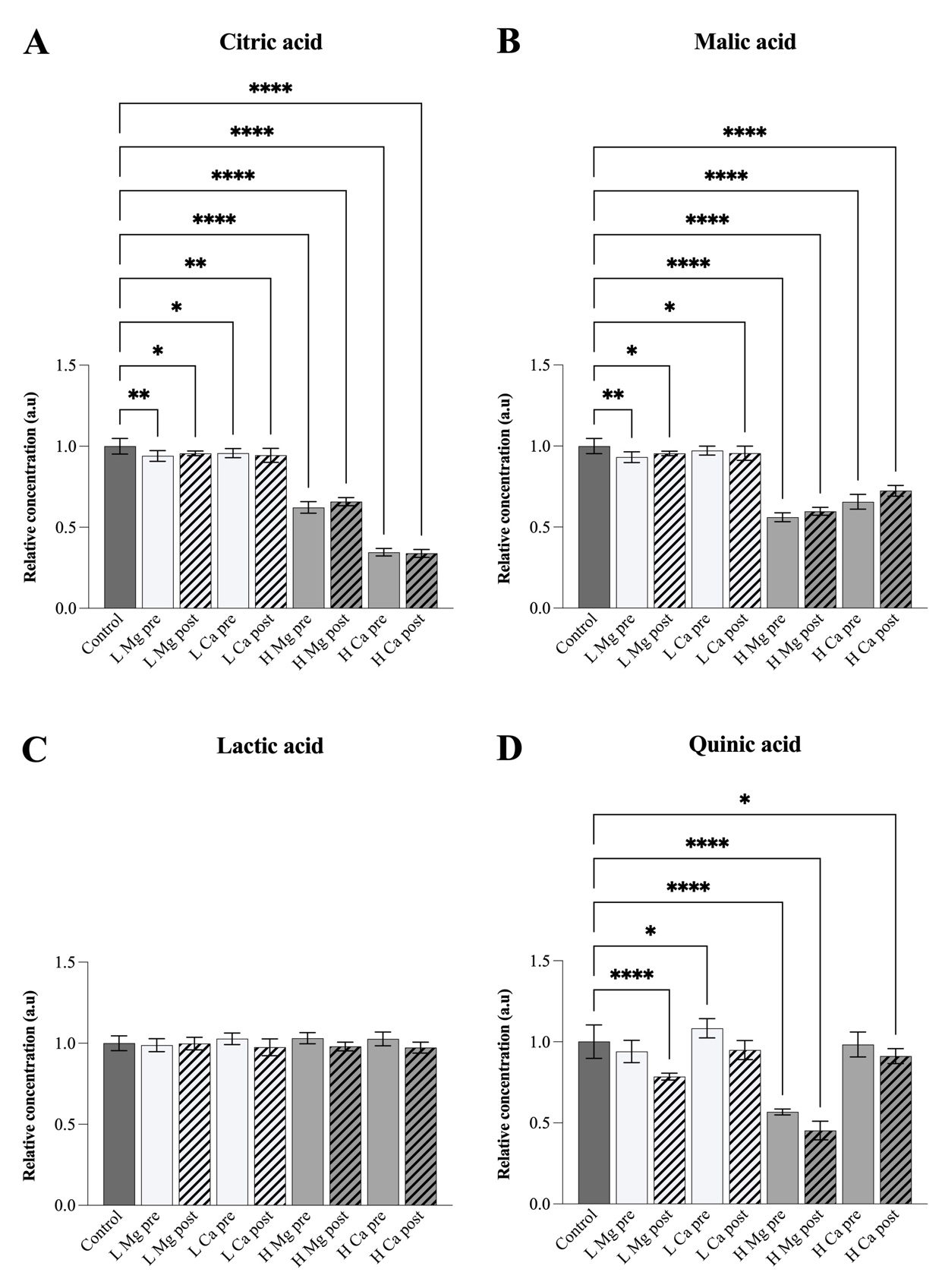


**Figure S3.** Relative concentration of selected acids as measured by GC-MS. The uncertainties are represented by the standard deviation of the mean (n=5). Low (100 ppm) and high (1000 ppm) salt concentrations are abbreviated L and H, respectively. Significant difference between treated sample and control is indicated. (* p < 0.05; ** p < 0.01; *** p < 0.001; **** p < 0.0001).


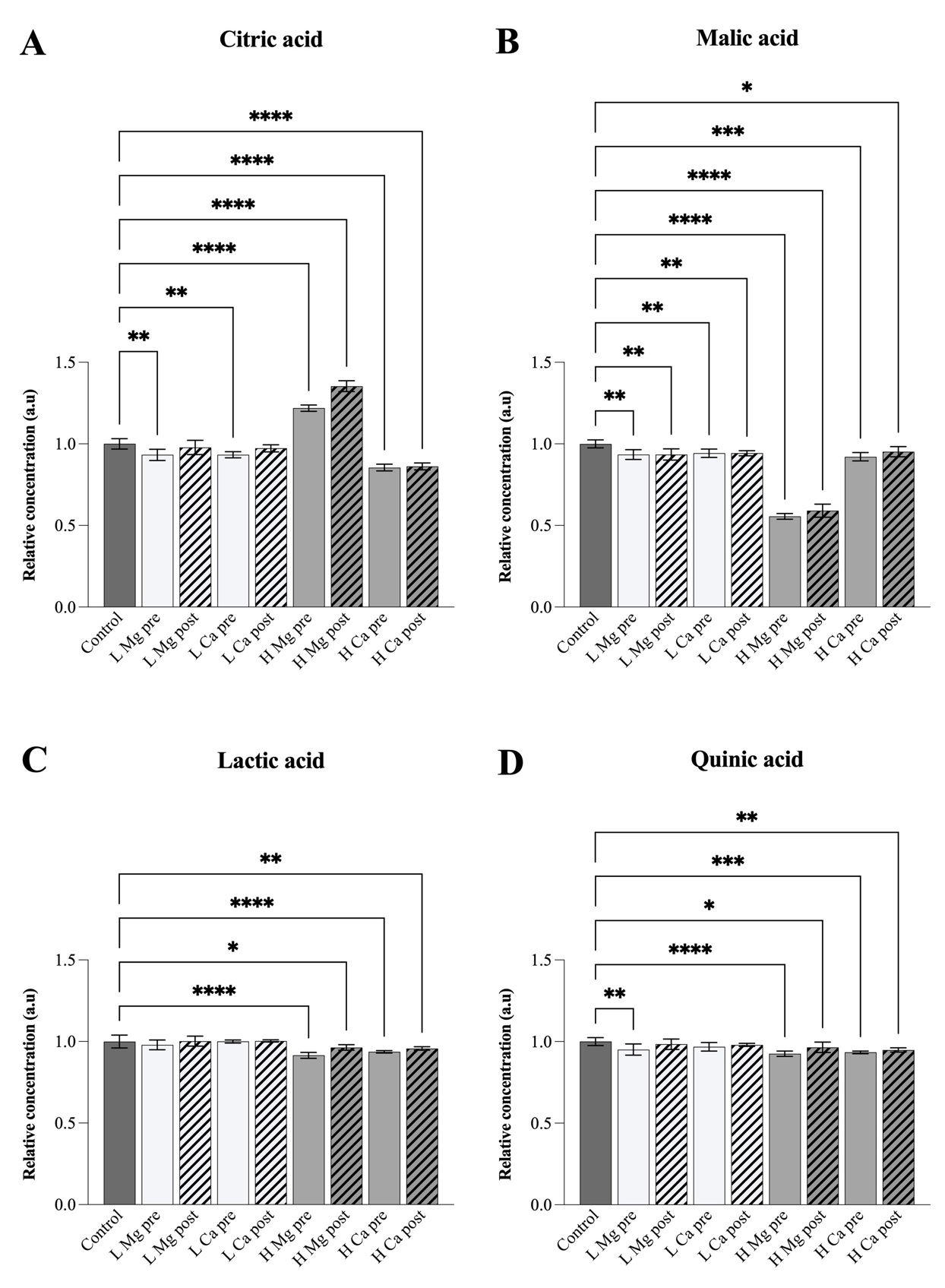


**Figure S4.** Relative concentration of selected acids as measured by NMR. The uncertainties are represented by the standard deviation of the mean (n=5). Low (100 ppm) and high (1000 ppm) salt concentrations are abbreviated L and H, respectively. Significant difference between treated sample and control is indicated. (* p < 0.05; ** p < 0.01; *** p < 0.001; **** p < 0.0001).


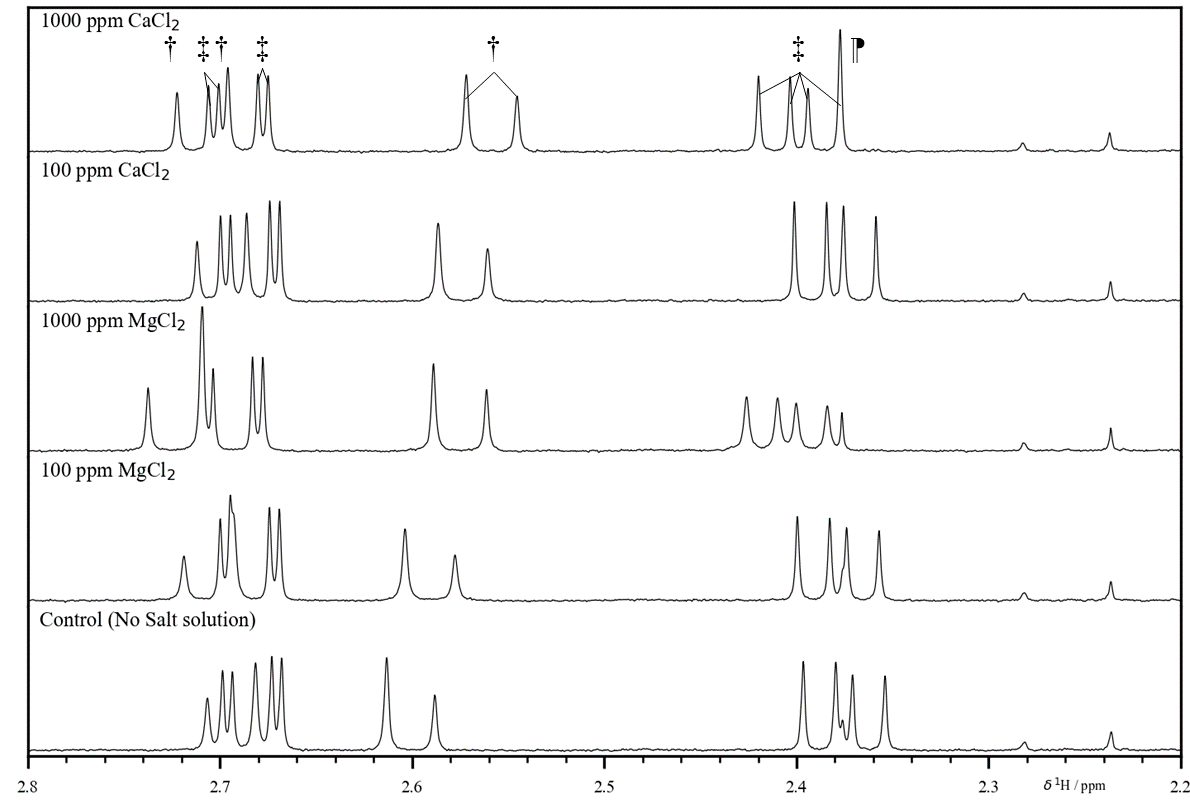


**Figure S5.** Selected region for the malic and citric acid peaks of the ^1^H spectra of the samples prepared to check for the effect of high salt concentration on the NMR quantification. Samples were left to equilibrate for 3 days in the NMR tube. *†*: Citrate peaks; *‡* Malate peaks; *⁋* Unknown contaminant.
